# Natural history of carotid artery free-floating thrombus—A single center, consecutive cohort analysis

**DOI:** 10.3389/fneur.2022.993559

**Published:** 2022-09-27

**Authors:** Mandy D. Müller, Nikolaos Raptis, Pasquale Mordasini, Werner Z'Graggen, Andreas Raabe, Philippe Schucht, Mirjam R. Heldner, David Bervini

**Affiliations:** ^1^Department of Neurosurgery, Inselspital, Bern University Hospital and University of Bern, Bern, Switzerland; ^2^Department of Neurology, University Hospital Basel, Basel, Switzerland; ^3^Department of Neuroradiology, Inselspital, Bern University Hospital and University of Bern, Bern, Switzerland; ^4^Department of Neurology, Bern University Hospital and University of Bern, Bern, Switzerland

**Keywords:** carotid free-floating thrombus, carotid endarterectomy, stroke risk, carotid thromboembolism, carotid thromboendarterectomy

## Abstract

**Introduction:**

Carotid free-floating thrombus (CFFT) is a rare cause of stroke and is thought to be associated with a high risk of recurrent cerebrovascular ischaemic events. The existing data on the natural history and optimal treatment modalities of CFFT is scanty and no clear recommendations exist.

**Objective:**

A retrospective analysis, single-center cohort of consecutive patients diagnosed with CFFT was conducted, investigating the risk for recurrent cerebrovascular ischaemic events.

**Methods:**

We performed a single-center retrospective analysis including all patients presenting at our tertiary center between January 2005 and December 2020 with symptoms consistent with ischaemic stroke and/or transient ischaemic attack. Digital subtraction angiography (DSA), computed tomography angiography (CTA) or magnetic resonance angiography (MRA) were used to diagnose CFFT. In all included patients, CFFT was confirmed with a second imaging modality. CFFT was defined on imaging as a defect in contrast filling extending into the carotid lumen. We gathered information on vascular risk factors, diagnosis and follow-up methods, modality of treatment and neurological outcome. A survival analysis was performed, assessing the risk for recurrent cerebrovascular events.

**Results:**

In total, *N* = 62 patients presenting with symptomatic CFFT were included. Mean age was 68 years, 69% (43/62) of patients were male, 52% (32/62) current or previous smokers, 76% (47/62) suffered from arterial hypertension, 68% (42/62) from dyslipidaemia, and 31% (19/62) from diabetes mellitus. Overall, 71% (44/62) of patients received any kind of intervention [endovascular or surgical carotid thrombo-endartectomy (CEA)] at any time point during follow-up. Sixteen percent of patients (10/62) received intervention within 48 h after diagnosis of CFFT. The survival analysis and Kaplan-Meier model censoring patients at the time of intervention or last follow-up showed that the risk for any recurrent ischaemic stroke was 19.7% within the first 7 days and 27.4% within 3 months after diagnosis. No patients experienced a new ischaemic stroke beyond 11 days after diagnosis of CFTT (*n* = 17).

**Conclusion:**

The risk of recurrent ischaemic events in patients with CFFT is high, especially in the first week after diagnosis. Prospective studies are needed to further investigate the optimal management of these patients.

## Introduction

Carotid free-floating thrombus (CFFT) is a rare cause of stroke. With the increased availability of imaging techniques such as computed tomography angiography (CTA) and magnetic resonance angiography (MRA) reports of this pathology have increased (1). The most common underlying etiology is atherosclerosis. However, CFFT has also been associated with hypercoagulable states such as malignancy, thrombophilia and inflammatory processes (2–4). Most patients present with acute neurological symptoms consistent with a transient ischaemic attack (TIA) or stroke. The risk for recurrent cerebrovascular events in these patients is thought to be particularly high (1, 5). A recent systematic literature review and meta-analysis identified >500 cases with CFFT from case series and case reports. The 30-day risk of TIA, silent brain ischaemia, or any stroke or death was estimated at 17.1% (1). However, optimal management remains unclear as high-quality evidence on the optimal treatment strategy is lacking. Controversy remains, whether these patients should be treated medically (heparin, antiplatelet agents) or receive interventional treatments such as carotid thrombo-endarterectomy (CEA), carotid artery stenting (CAS) or even mechanical thrombectomy. We therefore aimed to perform a retrospective analysis of all patients presenting with CFFT at our tertiary center summarizing information about their respective management (medical therapy and/or interventional therapy) and investigating the risk of recurrent cerebrovascular events.

## Methods

This single center retrospective cohort study was approved by the local ethics committee. All patients presenting at our tertiary center between January 2005 and December 2020 with acute neurological deficits consistent with acute ischaemic stroke and/or TIA in whom CFFT was diagnosed were included. CFFT was defined similar to previous reports as a contrast filling structure adhering to the carotid wall and extending into the lumen (1). Digital subtraction angiography (DSA), computed tomography angiography (CTA) or magnetic resonance angiography (MRA) were used to diagnose CFFT. We excluded patients in whom CFFT was not confirmed with a second radiological modality (CTA, MRA, duplex ultrasound). Moreover, patients receiving endovascular thrombectomy (EVT) due to intracranial large vessel occlusion at the time of diagnosis were excluded. Patients receiving EVT were excluded from our analysis because the indication for urgent endovascular treatment was intracranial large vessel occlusion rather than free-floating thrombus at the carotid bifurcation.

Data on vascular risk factors (arterial hypertension, dyslipidaemia, current/previous smoking, diabetes mellitus, and atrial fibrillation), demographic information, and information about the type of the index event were gathered for all patients. Patients presenting with acute neurological symptoms lasting <24 h without magnetic resonance imaging (MRI) evidence of acute cerebral ischaemia on diffusion-weighted imaging (DWI) sequences were classified as having a TIA. Patients with symptoms lasting >24 h and/or MRI evidence of acute cerebral ischaemia on DWI were classified as presenting with a stroke. Information on time point of treatment, type of treatment (conservatively vs. interventional including carotid endarterectomy and endovascular treatment), and of medications given were collected. Patients were followed clinically and with imaging.

On admission, patients were evaluated by a certified stroke physician using the NIH Stroke Scale (NIHSS). Functional outcome was measured using the modified Rankin scale (mRS) at admission, discharge and at follow-ups. Patients were routinely followed clinically and with duplex ultrasound. Recurrent cerebrovascular events were documented clinically and with follow-up imaging.

We performed a survival analysis and Kaplan-Meier model censoring patients at the time of treatment or at last follow-up. SPSS version 25.0 (IBM Corp, Chicago, IL) was used for all statistical analyses and an alpha level of 0.05 was defined to ascribe statistical significance.

## Results

**Figure 1:**
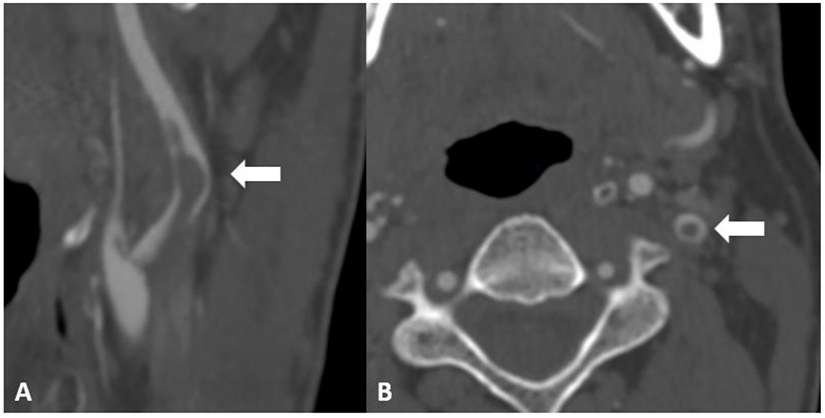
Computed tomography angiography image of a free-floating thrombus in the proximal left internal carotid artery. **(A)** depicts a sagittal view of the carotid bifurcation with a contrast filling defect corresponding to a free floating thrombus extending into the distal internal carotid artery lumen (arrow). **(B)** depicts an axial view of the internal carotid artery with a central contrast filling defect corresponding to the free-floating thrombus in the internal carotid artery.

Between January 2005 and December 2020, *N* = 74 patients in whom CFFT was diagnosed presented to our emergency department with acute neurological deficits consistent with acute ischaemic stroke and/or TIA. Twelve patients were excluded from analysis because they received endovascular carotid thrombectomy due to intracranial large vessel occlusion at the time of diagnosis. DSA, CTA or MRA were used to diagnose CFFT. In all included patients, the presence of a CFFT was confirmed with a second imaging modality. Figure 1 shows a free-floating thrombus in the proximal left internal carotid artery in a patient included in this analysis.

In total, we included *N* = 62 patients presenting with symptomatic CFFT in our analysis. Demographic information of our patient population is presented in Table 1. Overall, mean age was 68 years, 69% (43/62) of patients were male.

**Table 1:** Baseline characteristics of all (*N* = 62) patients included in the analysis.

Mean age (years, IQR)	68 (58–78)
Gender (male, %)	43/62 (69%)
**Vascular risk factors**	
Arterial hypertension (%)	47/62 (76%)
Dyslipidaemia (%)	42/62 (68%)
Diabetes mellitus (%)	19/62 (31%)
Current or previous smoking (%)	32/62 (52%)
Atrial fibrillation (%)	10/62 (16%)
**Medication on admission**	
Platelet inhibitors or oral anticoagulants (%)	28/62 (45%)
Statin (%)	19/62 (31%)
**Neurological status on admission**	
NIHSS (0–42) on admission (median, IQR)	4 (2–6)
mRS (0–6) on admission (median, IQR)	2 (1–3)
**Neurological status ad discharge**	
NIHSS (0–42) at discharge (median, IQR)	1 (0–3)
mRS (0–6) at discharge (median, IQR)	1 (0–2)

The majority of patients presented with an ischaemic stroke (90%, 56/62), only a minority presented with a TIA (10%, 6/62). On admission, 28 (45%) patients were on platelet inhibitor therapy or oral anticoagulant therapy. Length of thrombus was recorded in 33 of 62 patients. Mean length of thrombus was 21 mm (IQR 9.5–34.5 mm).

Forty-two patients (68%) received heparin in therapeutic doses as initial medical treatment, five (8%) received antiplatelet medication, and one patient was treated with intravenous thrombolysis (2%). One patient received no acute phase treatment and palliative care was initiated (2%). Thirteen patients received interventional treatment within 72 h of diagnosis (2/13 patients received CAS, 11/13 patients received CEA). Of these patients, 11 received antiplatelet therapy before treatment. Three patients received heparin additionally to antiplatelet therapy. Two patients received heparin only preoperatively.

In total, 71% (44/62) of patients received any kind of intervention (endovascular or CEA) at any time point during follow-up. Median time interval between diagnosis and interventional treatment was 5 days (IQR 3–9). Only 16% of patients (10/62) received intervention within 48 h after diagnosis of CFFT. Overall, 61% of patients (38/62) were treated with CEA at any time point.

Eleven (14%) patients experienced a new ischaemic stroke under best medical treatment without intervention. Nevertheless, the majority of these patients remained clinically stable reflected in an improved or stable NIHSS at discharge. Only one patient deteriorated clinically.

**Figure 2:**
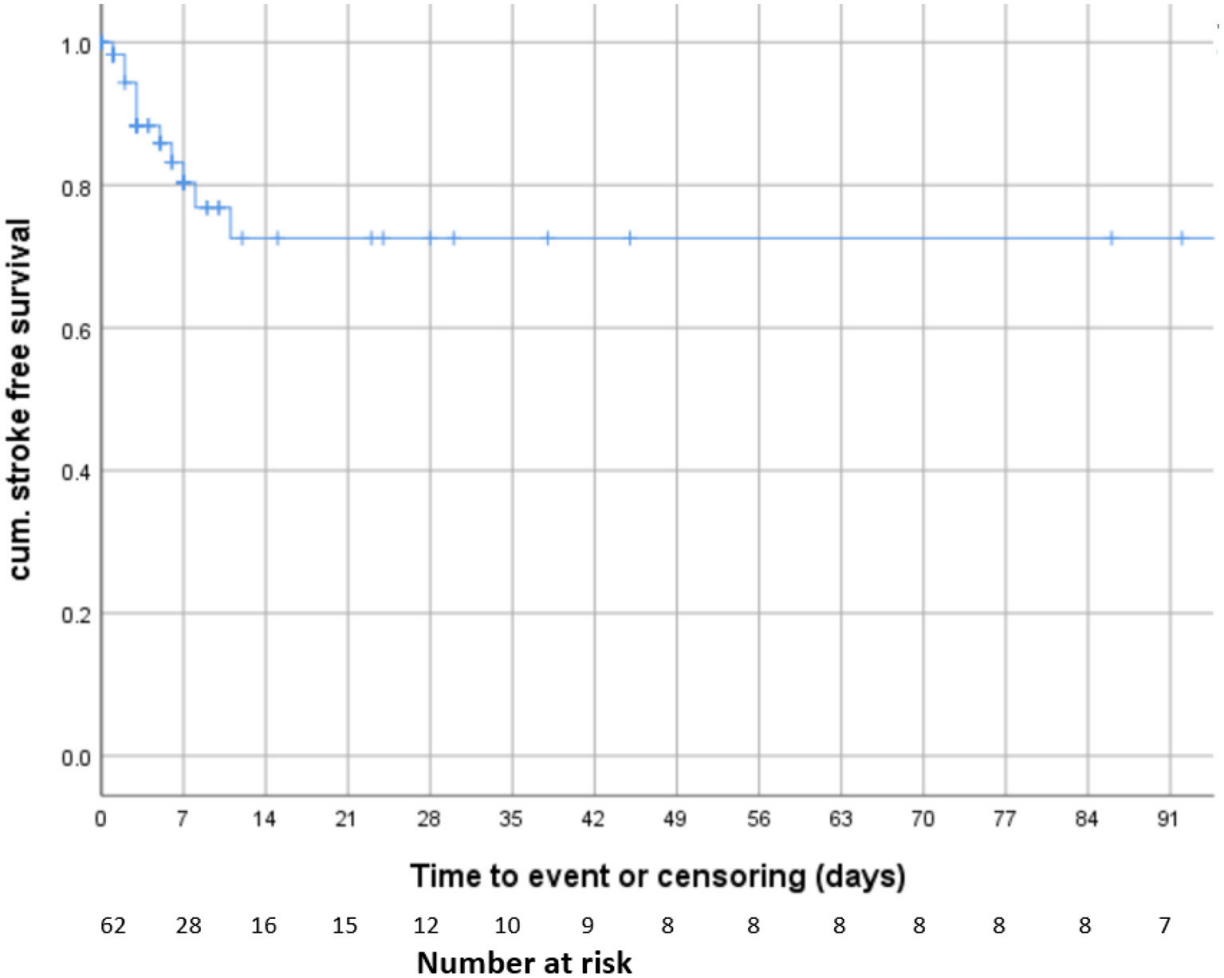
Survival analysis. Kaplan-Meier model including all patients and censoring patients at the time of treatment or last follow-up.

The survival analysis and Kaplan-Meier model, censoring patients at the time of treatment or last follow-up showed that the risk for recurrent ischaemic stroke was 19.7% within the first 7 days and 27.4% within 3 months after diagnosis (Figure 2). No patients experienced a new ischaemic stroke beyond 11 days after diagnosis of CFTT (*n* = 17, Figure 2).

## Discussion

This retrospective analysis of consecutive patients presenting with CFFT at our tertiary center showed that the risk of recurrent cerebrovascular events is very high, particularly in the first week after patients became symptomatic. These results concur with previous studies (1). To the best of our knowledge, this is the largest patients series published on patients with CFFT.

Evidence on the optimal treatment in these patients is currently lacking. To date, there has been no randomized or comparative trial investigating the risk associated with different treatment strategies for patients diagnosed with CFFT. No consensus exists whether these patients should be treated with heparin, antiplatelet agents, oral anticoagulation or a combination thereof. This uncertainty is also reflected in the heterogeneity of medical treatment patients in our study population received.

Furthermore, it remains controversial whether these patients benefit from interventional treatment (endovascular or surgical) in addition to medical treatment alone. Even more uncertainty remains concerning the optimal time point of CEA or CAS, if deemed appropriate. A recent meta-analysis reviewing 525 cases diagnosed with CFFT between 1960 and 2017 confirm the current uncertainty in best management of these patients. The authors also report highly heterogeneous medical therapies used in their series and a variety of interventional therapies. The authors conclude that neither medical nor interventional therapy is clearly superior and that prospective studies are needed to optimize treatment of these patients (1).

Because the risk for recurrent cerebrovascular events is especially high in the first few days after diagnosis, future treatment strategies should focus on lowering the risk of recurrent cerebrovascular events in the first few days after diagnosis. It remains uncertain, whether early CEA or CAS might lower the risk of recurrent ischaemic stroke in the acute phase. Several case reports showed that urgent CEA might be safe in these patients (2, 6, 7). However, evidence from prospective trials is currently lacking.

Due to the retrospective nature of this analysis and the heterogeneity of treatments patients received, a meaningful comparison of risks associated with different treatment strategies in our study population was not possible. Thus, our results have to be interpreted with caution. In order to optimize treatment for patients diagnosed with CFFT, prospective trials are needed.

## Conclusions

The risk of recurrent ischaemic event in patients with CFFT is high, especially in the first week after diagnosis. Prospective trials are needed to determine the optimal management of these patients.

## Data availability statement

The raw data supporting the conclusions of this article will be made available by the authors, without undue reservation.

## Ethics statement

The studies involving human participants were reviewed and approved by Kantonale Ethikkommission. Written informed consent for participation was not required for this study in accordance with the national legislation and the institutional requirements.

## Author contributions

All authors listed have made a substantial, direct, and intellectual contribution to the work and approved it for publication.

## Conflict of interest

The authors declare that the research was conducted in the absence of any commercial or financial relationships that could be construed as a potential conflict of interest.

## Publisher's note

All claims expressed in this article are solely those of the authors and do not necessarily represent those of their affiliated organizations, or those of the publisher, the editors and the reviewers. Any product that may be evaluated in this article, or claim that may be made by its manufacturer, is not guaranteed or endorsed by the publisher.
